# Nematocyst Types and Characteristics in the Tentacles of *Gershwinia thailandensis* and *Morbakka* sp. (Cubozoa: Carybdeida) from the Gulf of Thailand

**DOI:** 10.3390/biology13100845

**Published:** 2024-10-21

**Authors:** Thippawan Yasanga, Sineenart Santidherakul, Klintean Wunnapuk, Rochana Phuackchantuck, Lakkana Thaikruea, Thunyaporn Achalawitkun, Purinat Rungraung

**Affiliations:** 1 Medical Science Research Equipment Center, Faculty of Medicine, Chiang Mai University, Chiang Mai 50200, Thailand; sineenart.san@cmu.ac.th; 2 Research Administration Section, Faculty of Medicine, Chiang Mai University, Chiang Mai 50200, Thailand; rochana.p@cmu.ac.th; 3 Department of Forensic Medicine, Faculty of Medicine, Chiang Mai University, Chiang Mai 50200, Thailand; klintean.w@cmu.ac.th; 4 Consultancy of Working Group on Maritime Service Plan System Development, Region 11, Royal Thai Ministry of Public Health, Surat Thani 84000, Thailand; lakkana.t@cmu.ac.th; 5 Chumphon Fisheries Provincial Office, Department of Fisheries, Chumphon 86000, Thailand; thunya-flower@hotmail.com; 6 Marine and Coastal Resources Research Center, The Upper Gulf of Thailand, Department of Marine and Coastal Resources, Samut Sakhon 74000, Thailand; purinat03fuse@gmail.com

**Keywords:** box jellyfish, nematocysts, microbasic p-mastigophores, venom toxicity, cnidarians, morphology

## Abstract

Nematocysts, specialized stinging cells in cnidarians, play a crucial role in both defense and prey capture, containing venomous, coiled tubes within a capsule. While box jellyfish are recognized as a medical threat, information on the nematocysts of species like *Gershwinia thailandensis* and *Morbakka* sp. from Thai waters remains sparse. This study explores the types and morphology of nematocysts found in the tentacles of these species using light and scanning electron microscopy. We identified three nematocyst types: club-shaped microbasic p-mastigophores, oval isorhizas, and oval microbasic p-rhopaloids. Notably, significant differences in capsule sizes were observed, especially in the microbasic p-mastigophores and isorhizas. The discharge tubules tapered from the proximal to the distal ends, featuring arrow-shaped spines in a helical pattern. A distinct lancet structure was present in both microbasic p-mastigophores and p-rhopaloids. These findings suggest that variations in nematocyst size and morphology may be linked to evolutionary adaptations, functional roles, and venom toxicity. Further research into venom discharge mechanisms could offer valuable insights into the ecological and medical importance of these cnidarians.

## Simple Summary

This study investigated the nematocyst types found in the tentacles of box jellyfish *Gershwinia thailandensis* and *Morbakka* sp. in Thai waters, providing detailed taxonomic insights into these toxic stinging cells. Three nematocyst types were identified in both species: club-shaped microbasic p-mastigophores (Type 4), oval isorhizas, and oval microbasic p-rhopaloids. While the sizes of nematocyst capsules varied between species, the spination pattern of discharge tubules was consistent. These differences in nematocyst types, population densities, and morphologies may indicate evolutionary adaptations and functional specialization. Understanding these distinctions could aid in developing species-specific envenomation treatments and deepen our knowledge of the ecological and toxicological roles of these nematocysts. Further research on the molecular composition and venom release mechanisms will offer valuable biological insights.

## 1. Introduction

Currently, jellyfish in the phylum Cnidaria are categorized into three major classes: Scyphozoa (true jellyfish), Hydrozoa (hydroid jellyfish), and Cubozoa (box jellyfish). According to Collins [1], Cnidaria is monophyletic, consisting of Anthozoa and Medusozoa. While Cubozoa and Hydrozoa are well-supported clades, Scyphozoa appears paraphyletic. Cubozoa comprises two monophyletic orders: Chirodropida and Carybdeida [2,3,4,5]. Chirodropids have multiple tentacles per pedalium, while carybdeids possess only one. Chirodropids are known for their lethal venom, whereas carybdeid envenomations, though less dangerous, can still cause severe pain or be fatal [5,6]. As of 2022, 51 species of box jellyfish have been described [7].

Several species, including *Chironex indrasaksajiae* [8] and *Gershwinia thailandensis* [9], have been documented in Thailand, with at least 12 species recorded in Thai waters [10,11,12]. Surveys by the Thai Department of Marine and Coastal Resources (DMCR) identified five families of box jellyfish and two other venomous jellyfish species in the Andaman Sea and the Gulf of Thailand [13]. In Thailand, a report by Thaikruea (2023) [14] documented 124 cases of box jellyfish stings from 1999 to 2021, including 29 cases of single-tentacle box jellyfish (SBJ) and 92 cases of multi-tentacle box jellyfish (MBJ), with a fatality rate of 9.8% in the MBJ group. Despite being classified under the same category, SBJ and MBJ stings cause distinct clinical manifestations. SBJ stings typically lead to delayed symptoms, such as Irukandji syndrome and Irukandji-like syndrome, while MBJ stings result in immediate severe symptoms such as cardiotoxicity, dermatonecrosis, and hemolysis [14,15]. Additionally, some toxins from SBJ species, such as *Morbakka* spp., can cause immediate effects [16,17]. Box jellyfish use potent venom, which is stored in their stinging cells (cnidae), to quickly immobilize and kill their prey to prevent escape [18].

Cnidae, or cnidocytes, are specialized organelles unique to cnidarians [19,20], which are used for prey capture, defense, and habitat interaction. They are classified into three categories: nematocysts, spirocysts, and ptychocysts [21,22]. Despite their biological novelty, the developmental mechanisms driving cnidocyte diversity remain poorly understood [23].

Cnidae have been extensively studied across Hydrozoa, Scyphozoa, and Anthozoa [24,25,26,27,28,29,30,31] and are used as essential taxonomic tools in certain groups, such as Actiniaria [32,33]. Research into cnidae variability, such as in *Isactinia* sp. (Anthozoa) [31] and *Pelagia noctiluca* (Scyphozoa) [30], highlights their functional and taxonomic significance. Similarly, updated descriptions of species like *Cyanea nozakii* (Scyphozoa) [29] and *Lophelia pertusa* (Anthozoa) [28] emphasize their evolutionary importance across a range of cnidarians. Over 30 types of nematocysts have been described, primarily differing in the morphology of their basal spiny tubule, while spirocysts and ptychocysts exist in only one form each [23,34]. Nematocysts are key taxonomic tools in Cubozoa [4,5,35,36,37,38,39,40,41,42] and remain under-documented for many jellyfish species, particularly within Cubozoa. Gershwin (2006) [43] described the nematocysts of several Cubozoan species, such as *Malo maxima*, *Chiropsalmus quadrumanus*, *Morbakka*, and *Tripedalia binata*, primarily using light microscopy.

In cubozoans, tentacular nematocysts are arranged in transverse bands that are simple and uniform [44]. Nematocysts consist of a pressurized capsule containing a coiled tubule, which ejects upon stimulation to deliver toxins into the target and is crucial for prey capture or defense [45,46,47,48]. Harmful species cause intense pain, while harmless ones cause little to no discomfort [49].

Nematocyst identification is based on the morphology of discharged capsules, focusing on the ratio of the length of the shaft to the length of the capsule, the position of shaft swellings, and the location of spines [19,43,50,51,52]. Common nematocyst types in cubozoans include euryteles, mastigophores, and isorhizas, each differing in shaft characteristics [43].

Research has explored the relationship between nematocyst types, venom complexity, and toxicity. For example, studies on *Chironex fleckeri* showed that microbasic mastigophores likely contain toxins, while small atrichous isorhizas and microbasic euryteles did not exhibit toxic activity [53]. Further studies separated different nematocyst types and identified distinct protein extracts in *Chironex fleckeri* tentacles [54].

Building on these insights, the aim of this study was to examine the tentacular nematocysts of *Gershwinia thailandensis* and *Morbakka* sp. (referred to as *Morbakka* sp. A) from the Gulf of Thailand. Using light and scanning electron microscopy, we aimed to provide detailed insights into nematocyst structures and their relationships within Cubozoa and across Cnidaria.

## 2. Materials and Methods

### 2.1. Specimen Collection

Specimen collections were conducted using methods adapted from Thaikruea and Santidherakul [55] and Yanagihara et al. [40]. We collected specimens of single-tentacled box jellyfish, *Gershwinia thailandensis*, from beaches in the Gulf of Thailand, specifically at Narathat Beach and Tak Bai (Narathi-wat Province) and Laem-ta-chee Beach (Pattani Province). *Morbakka* sp. (referred to as *Morbakka* sp. A) was collected from Chaloklum Beach (Koh Pha Ngan, Surat Thani Province). Animal taxonomic identification was performed by trained staff from the Marine and Coastal Resources Research Center. Tentacle samples were collected for analysis, and the whole animals were subsequently preserved and stored for future reference.

Tentacle samples were excised (1–2 cm per piece) from the tips to the middle regions, depending on the availability of the full structure. The tentacles were placed in distilled water for 1–2 min to induce nematocyst discharge and then transferred to a fixative containing 2.5% glutaraldehyde in 0.1 M PBS (pH 7.4). The samples were stored at 4 °C. After fixation, the tentacles were rinsed with PBS and observed using scanning electron microscopy (SEM). The remaining fixative solution was centrifuged (3000× *g*, 2 min), and the supernatant was removed. The nematocysts were then rinsed with double-distilled water for further analysis.

### 2.2. Analyses and Classification of Isolated Nematocysts Using Light Microscopy

For nematocyst analysis and identification, isolated nematocysts were suspended in a small volume of double-distilled water (DDW). A drop of the suspension was placed directly onto a microscope slide and examined using a BX63 Olympus microscope (Tokyo, Japan). The length and width of the undischarged nematocysts were measured as straight lines; the length was measured from the posterior to the anterior end of the capsule, and the width was measured perpendicularly across the widest point of the capsule, excluding any curvature. Both discharged and undischarged nematocysts were analyzed for their morphological features using a combination of light microscopy and scanning electron microscopy (SEM). The identification of nematocyst types in this study follows the nomenclature scheme outlined by Gershwin (2006) [43], which is detailed as follows:

1. Club-shaped microbasic p-mastigophores: A type of nematocyst in which the length of the discharged shaft does not exceed that of the capsule. The undischarged form features a distinct V-shaped notch at the distal end of the shaft, while the discharged shaft has long spines oriented away from the capsule (mastigophores Type 4).
2. Oval isorhizas: A type of nematocyst without a well-defined shaft. The discharged tubule has a uniform diameter throughout its length or narrows slightly toward the distal end.
3. Oval microbasic p-rhopaloids: A type of nematocyst in which the discharged shaft length does not exceed that of the capsule. It has a V-shaped notch in the undischarged shaft, similar to p-mastigophores, but the discharged shaft has a lobed appearance.

### 2.3. Scanning Electron Microscopy

Scanning electron microscopy was performed following methods adapted from Yanagihara et al. [40]. Excised tentacles were placed in distilled water for 1–2 min to induce nematocyst discharge and placed in a fixative containing 2.5% glutaraldehyde in 0.1 M PBS for at least 1 h at 4 °C and then post-fixed in 1% osmium tetroxide in double distilled water for another hour. Afterward, the samples were dehydrated through a graded ethanol series (50%, 70%, 85%, 95%, and 100%) and dried using a Quorum K850 Critical Point Dryer (Quorum Technologies, East Sussex, UK). Once dried, the samples were mounted on aluminum stubs and sputter-coated with gold using a Quorum Q150R S (Quorum Technologies, East Sussex, UK). Observations and photographs were taken using a JEOL JSM 6610LV scanning electron microscope (JEOL, Tokyo, Japan).

### 2.4. Statistical Analysis

The dimensions (length and width) of undischarged nematocyst capsules were measured from light microscopy images. The normality of the length and width data for all nematocyst types was assessed using the Shapiro–Wilk and Kolmogorov–Smirnov tests, as recommended by Acuña et al. [32,33] and Garese et al. [56]. Due to the non-normal distribution of several groups, a Kruskal–Wallis test was subsequently applied. Data were presented as mean ± standard deviation (SD) and range (minimum-maximum values) and were followed by Dunn’s test for multiple comparisons. A *p*-value of <0.01 was considered statistically significant. All statistical analyses were performed using IBM SPSS Statistics version 22.0 (IBM Corp., 2013, Armonk, NY, USA) for Windows. In this study, due to limitations in obtaining high-quality samples, cnidae measurements for *Morbakka* sp. A were derived from a single individual. In contrast, measurements for *Gershwinia thailandensis* were collected from multiple individuals, providing a representative range across specimens.

## 3. Results

### 3.1. General Nematocyst Types in Gershwinia thailandensis and Morbakka sp. A

This study presents the first detailed report on the tentacular nematocysts of single-tentacled box jellyfish found in Thai waters. Three distinct types of tentacular nematocysts were identified in *Gershwinia thailandensis* and *Morbakka* sp. A: club-shaped microbasic p-mastigophores (Type 4), oval isorhizas, and oval microbasic p-rhopaloids.

#### 3.1.1. Nematocyst Types in the Tentacles of *Gershwinia thailandensis*

**Figure 1:**
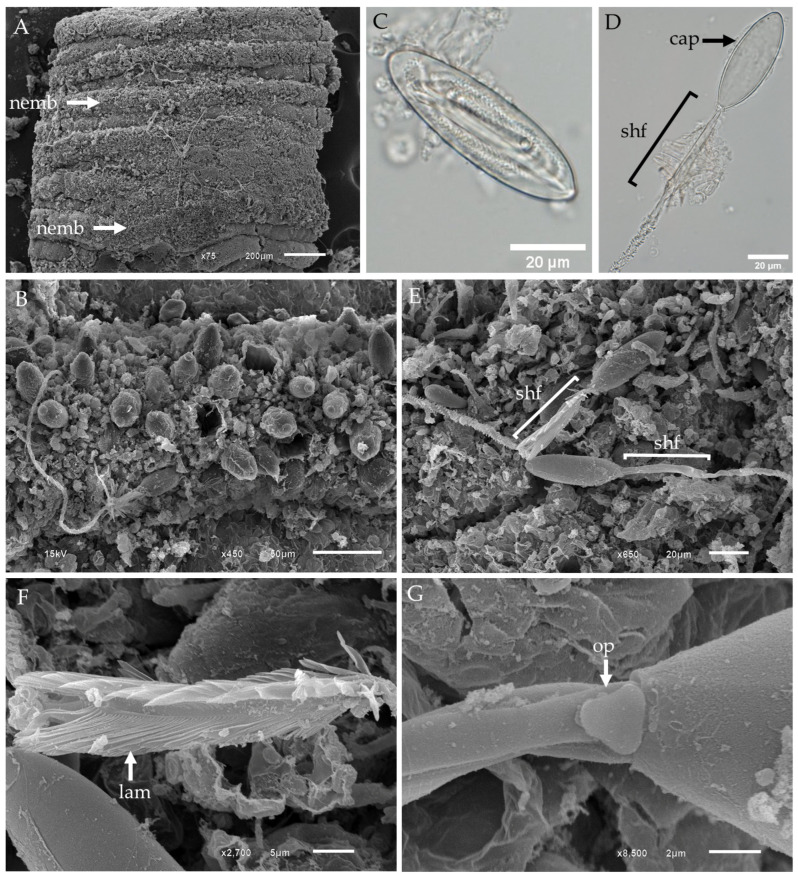
LM and SEM of *Gershwia thailandensis* tentacle and tentacular nematocysts. (**A**) Low-magnification SEM of the tentacle. (**B**) High-magnification SEM of the tentacle surface reveals a cluster of undischarged and discharged nematocysts. (**C**) LM of an isolated undischarged club-shaped microbasic p-mastigophore. (**D**) LM of discharged club-shaped microbasic p-mastigophore. (**E**) SEM of discharged club-shaped microbasic p-mastigophores shows well-released spine shafts and an absence of intact spine shafts. (**F**) SEM showing an enlarged view of the spine shaft of a microbasic p-mastigophore. Note the spine shaft, also called lamellae. (**G**) SEM of an incompletely discharged club-shaped microbasic p-mastigophore showing the absence of a spine shaft and the presence of an operculum. Abbreviations: cap, capsule; lam, lamellae; nemb, nematocyst band; op, operculum; shf, shaft.

In this study, three types of tentacular nematocysts were observed in *Gershwinia thailandensis* (Figure 1). Low-magnification SEM micrographs of the fixed tentacles revealed alternating bands of undischarged nematocysts and areas devoid of nematocysts arranged on the outer surface of the tentacle (Figure 1A). Higher-magnification SEM images showed both undischarged and discharged nematocysts (oval microbasic p-rhopaloids) with evaginated shafts and tubules (Figure 1B).

The tentacles of *Gershwinia thailandensis* were predominantly populated by club-shaped microbasic p-mastigophores (Type 4) (Figure 1C–G). Undischarged club-shaped microbasic p-mastigophores exhibited a shaft with a V-shaped notch at the distal end and a coiled tubule inside the capsule (Figure 1C). Light microscopy (LM) images of discharged club-shaped microbasic p-mastigophores revealed empty capsules, spine shafts, and spine tubules (Figure 1D). Both fully and partially discharged nematocysts were observed; fully discharged nematocysts had well-preserved spine shafts, while partially discharged nematocysts did not display visible spine shafts (Figure 1E).

SEM micrographs of the enlarged spine shafts of the club-shaped microbasic p-mastigophores showed that the stratified shafts had broad lamellae, with spines oriented away from the capsule (Figure 1F). Based on the classification by Gershwin [43], these nematocysts were identified as Type 4 p-mastigophores. In contrast, high-magnification SEM images of incompletely discharged nematocysts showed no spines attached to the shaft area, and a triangular-shaped operculum was visible at the base of the capsule (Figure 1G).

**Figure 2:**
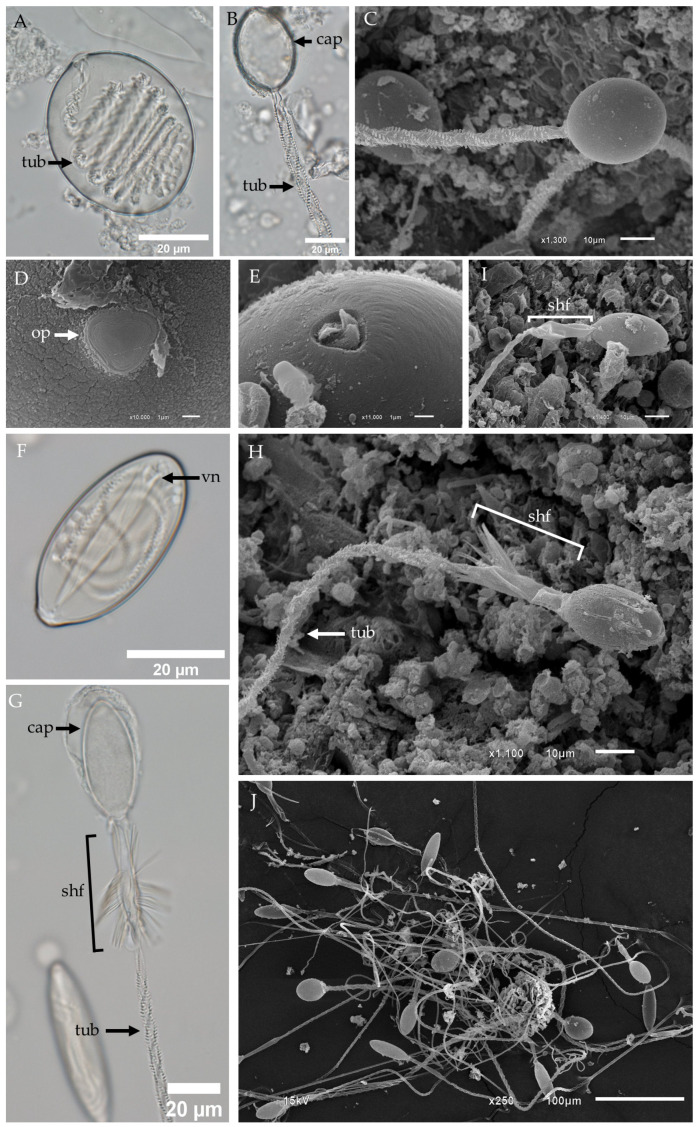
LM and SEM images of oval isorhizas and oval microbasic p-rhopaloids in the tentacle of *Gershwinia thailandensis*. (**A**) LM of an undischarged oval isorhiza. (**B**) LM and (**C**) SEM of a completely discharged oval isorhiza. Note that the discharged isorhiza shows an empty capsule and a tubule armed with spines arranged in a helical pattern. (**D**) SEM showing the top view of an oval isorhiza, highlighting the operculum. (**E**) SEM showing the top view of an oval isorhiza, with the operculum absent and the tip of the tubule visible, which is the first part to be expelled from the capsule. (**F**) LM of an undischarged oval microbasic p-rhopaloid. (**G**) LM and (**H**) SEM of a discharged oval microbasic p-rhopaloid, showing the spines on the shaft. Highlighting the distinctive whorl pattern of spines along the shaft. (**I**) SEM of a discharged oval microbasic p-rhopaloid, showing a single dilated shaft without spines. (**J**) SEM of various discharged tentacular nematocysts. Abbreviations: cap, capsule; op, operculum; shf, shaft; tub, tubule; vn, V-shaped notch.

In *Gershwinia thailandensis*, oval isorhizas are also distributed within the tentacle tissue (Figure 2A–E). Figure 2A shows an LM micrograph of an undischarged oval isorhiza, revealing its characteristic oval capsule shape with a coiled tubule inside. Fully discharged oval isorhizas exhibit empty capsules and tubules covered with spines arranged in a helical pattern (Figure 2B,C). The morphological details of the oval isorhiza’s operculum were further examined using SEM, which revealed its triangular shape (Figure 2D). As described by Östman [19], the tubule in most isorhizas begins to form coils near the aperture, looping back perpendicularly from wall to wall. In this study, the initial stage of discharge in the oval isorhiza shows that the operculum is absent, with the tip of the tubule being the first part to evaginate from the capsule. Interestingly, the tip resembles a propeller with three twisted blades (Figure 2E).

The third type of nematocyst found in the tentacles of *Gershwinia thailandensis* is the oval microbasic p-rhopaloid (Figure 2F–I). An LM micrograph of the undischarged oval microbasic p-rhopaloid reveals an intact capsule, with a V-notched shaft end and a coiled tubule inside (Figure 2F). When discharged, the LM image shows a spineless proximal shaft that is narrower than the distal shaft, which becomes dilated and is equipped with prominent spines (Figure 2G). In the fully discharged state, as observed through SEM (Figure 2H), the spine shaft structure appears misaligned, and some discharged oval microbasic p-rhopaloids lack spine shafts, with dilated distal shafts visible (Figure 2I). The cluster of fixed discharged tentacular nematocysts in *Gershwinia thailandensis* shows a substantial number of incomplete discharges, in which spine shafts and tubules are not fully exhibited (Figure 2J).

#### 3.1.2. Nematocyst Types Found in Tentacle of *Morbakka* sp. A

**Figure 3:**
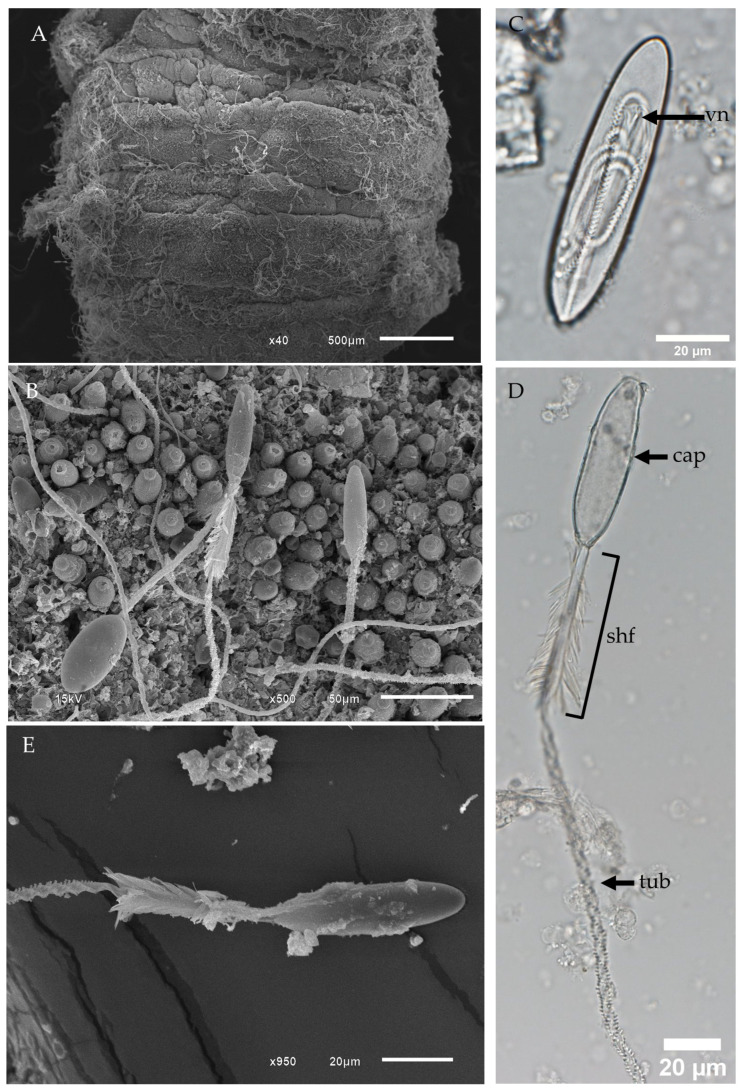
Light micrographs (LM) and scanning electron micrographs (SEM) of *Morbakka* sp. A tentacle and tentacular nematocysts. (**A**) Low-magnification SEM of the tentacle surface. (**B**) High-magnification SEM showing undischarged and discharged nematocysts on the tentacle surface. (**C**) LM of an undischarged club-shaped microbasic p-mastigophore. (**D**) LM and (**E**) SEM of a fully discharged club-shaped microbasic p-mastigophore, displaying the capsule, shaft, and tubule. Note the orientation of the spines away from the capsule. Abbreviations: cap, capsule; shf, shaft; tub, tubule; vn, V-shaped notch.

**Figure 4:**
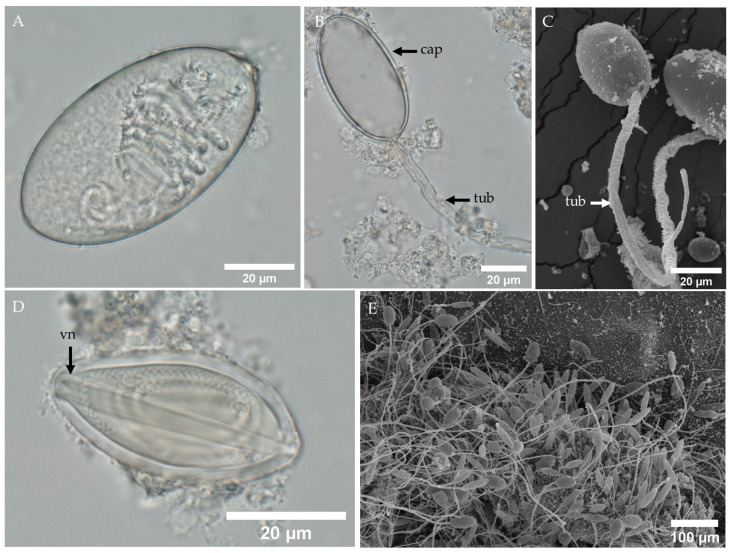
LM and SEM of oval isorhizas and oval microbasic p-rhopaloids in the tentacle of *Morbakka* sp. A. (**A**) LM of an isolated undischarged large oval isorhiza. (**B**) LM and (**C**) SEM of a discharged large oval isorhiza. Note the partially discharged proximal tubule remains encased in a membrane. (**D**) LM of an oval microbasic p-rhopaloid, with observed in small numbers. (**E**) SEM at low magnification of tentacular nematocysts reveals that club-shaped microbasic p-mastigophores are the dominant type, followed by large oval isorhizas, with a notable absence of microbasic p-rhopaloids. Abbreviations: cap, capsule; tub, tubule; vn, V-shaped notch.

The tentacles of *Morbakka* sp. A are primarily populated by club-shaped microbasic p-mastigophores (Type 4), followed by large oval isorhizas, with a smaller number of oval microbasic p-rhopaloids (Figure 3 and Figure 4). Initially, scanning electron microscopy (SEM) of the fixed tentacles at low magnification revealed alternating bands of undischarged nematocysts on the outer surface of the tentacle interspersed with areas devoid of nematocysts (Figure 3A). Upon closer examination, the tentacle surface displayed both undischarged and discharged nematocysts (Figure 3B).

The most abundant nematocyst type in *Morbakka* sp. A is the club-shaped microbasic p-mastigophores (Type 4) (Figure 3C,D). Undischarged microbasic p-mastigophores exhibit a shaft and a coiled tubule within the capsule (Figure 3C). Upon full discharge, the capsule becomes empty, revealing a spine shaft and tubule (Figure 3D,E). The spine shaft in this type of nematocyst is oriented away from the capsule, which is characteristic of Type 4 p-mastigophores (Figure 3E).

Other types of tentacular nematocysts found in *Morbakka* sp. A include large oval isorhizas (Figure 4A–C). The LM of the undischarged large oval isorhiza shows a tightly coiled tubule inside the capsule (Figure 4A). The LM (Figure 4B) and SEM (Figure 4C) images of the discharged large oval isorhiza reveal an empty capsule with a tubule. In Figure 4C, the discharged large oval isorhiza shows spines of the proximal tubule still partially encased by a membrane, while the spines from the proximal to middle regions are fully exposed.

This study also identified a small population of oval microbasic p-rhopaloids (Figure 4D), although no discharged oval microbasic p-rhopaloids were observed. At low magnification, SEM confirmed that the predominant type of tentacular nematocyst in *Morbakka* sp. A is the club-shaped microbasic p-mastigophores (Figure 4E).

### 3.2. Variation of Nematocyst Size Between the Species

In this study, we measured the length and width of tentacular nematocyst capsules using light microscopy, with the results presented in Table 1. Comparisons were made among the same types of nematocysts across the two species. The results showed that the club-shaped microbasic p-mastigophores in *Morbakka* sp. A (71.04 ± 10.30 µm) were the longest, followed by those in *Gershwinia thailandensis* (58.25 ± 6.56 µm). The difference in length between these two species was statistically significant (*p* < 0.01). However, there was no significant difference in the width of club-shaped microbasic p-mastigophores between *Morbakka* sp. A (17.49 ± 2.40 µm) and *Gershwinia thailandensis* (17.07 ± 1.80 µm).

For oval isorhizas, *Morbakka* sp. A exhibited the largest size, with a mean length of 72.74 ± 7.10 µm and a mean width of 38.58 ± 3.47 µm. The length measurements were significantly different (*p* < 0.01) compared to those of *Gershwinia thailandensis* (length: 45.71 ± 5.33 µm, width: 34.98 ± 3.44 µm). However, there were no significant differences in width between the two species (see Table 1 for detailed statistical values).

Regarding oval microbasic p-rhopaloids, the measurements were as follows: *Gershwinia thailandensis* had a length of 44.28 ± 2.45 µm and a width of 22.19 ± 1.69 µm, while *Morbakka* sp. A had a length of 42.74 ± 6.06 µm and a width of 19.56 ± 1.33 µm. No significant differences in this nematocyst type were found between the species (*p* > 0.01) (Table 1).

### 3.3. Tubule Morphology and Characteristics of the Spine of Discharged Tentacular Nematocysts of Gershwinia thailandensis and Morbakka sp. A

**Figure 5:**
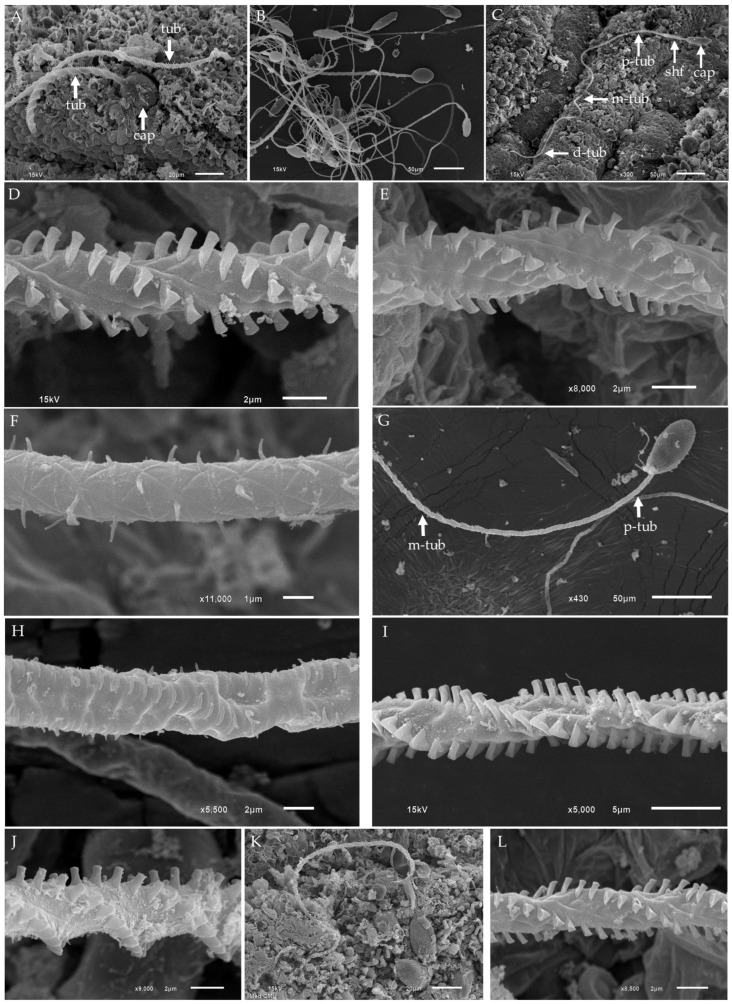
SEM images of tentacular nematocyst tubules from *Gershwinia thailandensis* and *Morbakka* sp. A. (**A**,**B**) Low-magnification SEM of discharged oval isorhiza and club-shaped microbasic p-mastigophore tubules from *Gershwinia thailandensis*. The tubule diameter of the oval isorhiza is notably larger compared to other nematocyst types. (**C**) SEM of a discharged club-shaped microbasic p-mastigophore from *Gershwinia thailandensis*, showing a gradual decrease in tubule diameter from the proximal to the distal end. (**D**) High-magnification SEM of the proximal region of the tubule from a discharged club-shaped microbasic p-mastigophore, highlighting the broader diameter. (**E**) High-magnification SEM of the middle region of the tubule from a discharged club-shaped microbasic p-mastigophore. (**F**) High-magnification SEM of the distal region of the tubule from a discharged club-shaped microbasic p-mastigophore, showing smaller spines and a significantly narrower tubule diameter. (**G**) Low-magnification SEM of a discharged oval isorhiza from *Morbakka* sp. A. (**H**) High-magnification SEM of an incompletely released spine on the proximal tubule of a discharged oval isorhiza. (**I**) High-magnification SEM of a fully released spine on the tubule of an oval isorhiza, showing an arrow-shaped spine arranged in a helical pattern in the middle region. (**J**) Enlarged SEM of the tubule of a discharged oval isorhiza from *Gershwinia thailandensis*, armed with arrow-shaped spines. (**K**) Low-magnification SEM of a discharged oval microbasic p-rhopaloid from *Gershwinia thailandensis*. (**L**) High-magnification SEM of the tubule from a discharged oval microbasic p-rhopaloid, showing arrow-shaped spines along the tubule. Abbreviations: d-tub, distal tubule; cap, capsule; m-tub, middle tubule; p-tub, proximal tubule; shf, shaft; tub, tubule.

In this study, the three types of tentacular nematocysts—club-shaped microbasic p-mastigophores, oval isorhizas, and oval microbasic p-rhopaloids—observed in *Gershwinia thailandensis* and *Morbakka* sp. A displayed differences in capsule shape and shaft characteristics. However, all well-preserved, fully discharged nematocysts demonstrated a consistent pattern of decreasing tubule thickness, spine shape, and spine arrangement (Figure 1D,E, Figure 2B,C,G,H, Figure 3D and Figure 4C). In this section, we focus on the detailed characteristics of the tubule and spines of the tentacular nematocysts, utilizing SEM for a clearer visualization of the spines. SEM micrographs of the tentacle surface in *Gershwinia thailandensis* showed that the discharged tubules of isorhizas had a larger diameter compared to the club-shaped microbasic p-mastigophores. However, the helical patterns of spine arrangement were noticeably similar (Figure 5A,B). The fully discharged club-shaped microbasic p-mastigophores in *Gershwinia thailandensis* exhibited tubules covered with spines arranged in a right-handed helical pattern, with the tubule thickness decreasing gradually from the proximal to the distal end (Figure 5C). To provide a clearer view of the spines along the tubule, enlarged SEM images of the proximal, middle, and distal regions of the discharged club-shaped microbasic p-mastigophore are shown in Figure 5D–F, respectively. The proximal (Figure 5D) and middle regions (Figure 5E) of the tubule are armed with broad-based, arrow-shaped spines with wide, blunt tips. In contrast, the distal region (Figure 5F) has a narrower tubule and is equipped with smaller spines.

The discharged tubules of oval isorhizas from all species in this study showed similar characteristics under SEM. The tubule thickness gradually decreases from proximal to distal regions, with broad-based, arrow-shaped spines arranged in a right-handed helical pattern. In some cases, the discharge of oval isorhizas in *Morbakka* sp. A showed incomplete release of spines at the proximal tubule, in which some spines remained trapped within the membrane, while spines in subsequent regions were fully released (Figure 5G). High-magnification SEM of discharged oval isorhiza tubules showed spines still trapped within the membrane (Figure 5H), whereas, in the middle region, well-released spines exhibited broad bases and arrow-shaped tips and were arranged in a right-handed helical pattern (Figure 5I,J).

For oval microbasic p-rhopaloids, the tubule and spine characteristics were similar to those observed in the other nematocyst types (Figure 5K,L).

### 3.4. Characteristic of Lancet Structure in Tentacular Nematocysts of Gershwinia thailandensis and Morbakka sp. A

According to Karabulut et al. [47], the compressed shaft structure of cnidarian nematocysts consists of densely coiled filaments that are vertically oriented relative to the capsule aperture and organized into stacked lamellae. Their study outlined three principal phases of nematocyst operation: (1) shaft discharge, (2) shaft eversion, and (3) tubule eversion. During these phases, the compressed shaft transitions into a loose, triple-helical structure as it undergoes eversion, and the tip of the everted shaft often forms a spearhead-like structure. These findings provided significant new insights into the early structural transformations of nematocysts during discharge.

**Figure 6:**
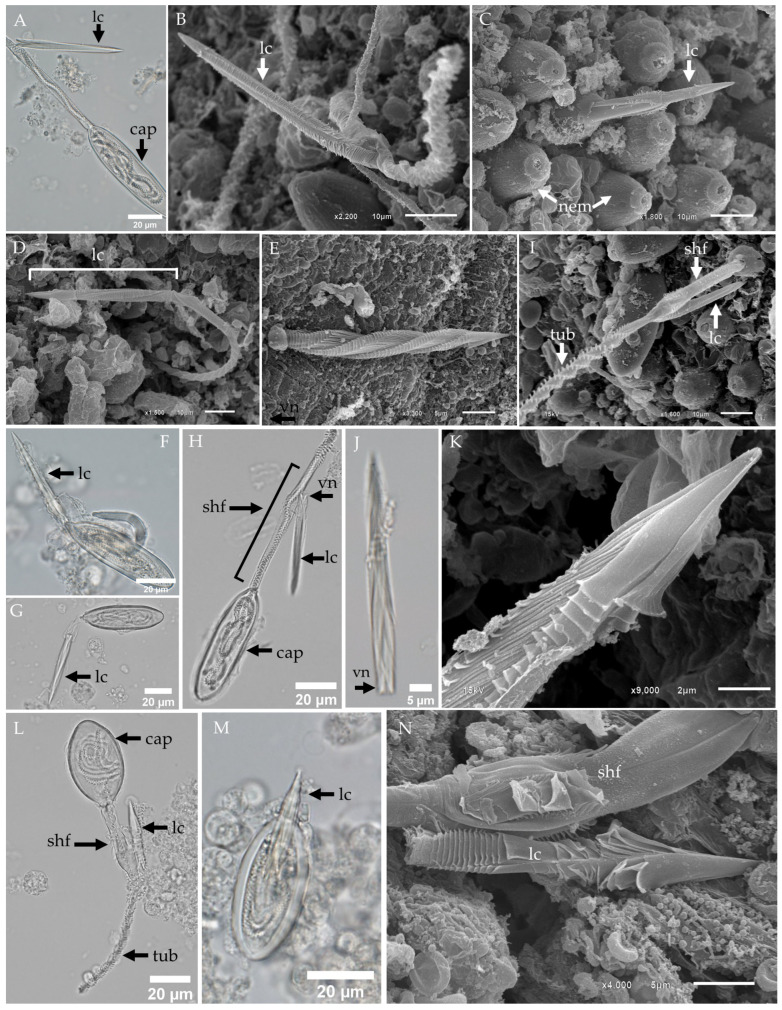
LM and SEM of the lancet structure in nematocysts of *Gershwinia thailandensis* and *Morbakka* sp. A. (**A**) LM and (**B**) SEM of the discharged club-shaped microbasic p-mastigophore from *Morbakka* sp. A showing the lancet attached near the shaft before the tubule begins. (**C**) SEM of the initial discharge of a club-shaped microbasic p-mastigophore from *Morbakka* sp. A indicated by the appearance of the lancet from the capsule on the tentacle surface. (**D**,**E**) SEM images of the entire lancet observed on the surface of a fixed tentacle from *Gershwinia thailandensis*. Note the length of the shaft, which corresponds to the club-shaped microbasic p-mastigophore. (**F**,**G**) LM images showing the everted lancet from the capsule of a microbasic p-mastigophore from *Gershwinia thailandensis*. (**H**,**I**) LM and SEM of a discharged club-shaped microbasic p-mastigophore from *Gershwinia thailandensis* with the lancet attached. (**J**) LM of a free V-notched lancet from a microbasic p-mastigophore of *Gershwinia thailandensis*. (**K**) SEM showing an enlarged view of the tip of the lancet from a microbasic p-mastigophore of *Gershwinia thailandensis*. (**L**) LM of a discharged oval microbasic p-rhopaloid from *Gershwinia thailandensis* with the lancet attached. (**M**) LM of the initial discharge of a microbasic p-rhopaloid from *Gershwinia thailandensis*, indicated by the appearance of the lancet from the capsule. (**N**) High-magnification SEM of the lancet attached to the shaft of a discharged microbasic p-rhopaloid from *Gershwinia thailandensis*. Abbreviations: cap, capsule; lc, lancet; nem, nematocyst; shf, shaft; tub, tubule; vn, V-shaped notch.

In our study, discharged club-shaped microbasic p-mastigophores and oval microbasic p-rhopaloids occasionally retained an intact rod-like filament, referred to as a lancet, as previously characterized by Yanagihara et al. [40]. This lancet remained contiguous, with the main shaft following release from the capsule. However, in the majority of fixed discharged nematocysts, the lancet was absent or not clearly visible under both light microscopy (LM) and scanning electron microscopy (SEM). Only a limited number of samples exhibited the lancet still attached to the shaft (Figure 6A–N).

Karabulut et al. [47] postulated that the helical configuration of the nematocyst shaft filaments serves as a structural mechanism that uncoils during discharge, potentially contributing to the rapid force generation required for efficient puncture and penetration of prey tissues. Our observations of the lancet structure support their conclusion that these filaments likely represent tightly packed helical elements, which transition into an elongated, spear-like form during discharge. In discharged club-shaped microbasic p-mastigophores from *Morbakka* sp. A, LM and SEM revealed that the lancet, which is characterized by a V-shaped notch at its terminal end, was still attached to the shaft during early discharge stages, while the tubule had not yet fully everted from the capsule (Figure 6A). This suggests that the lancet could play a stabilizing role in the initial stages of shaft eversion, which is consistent with Karabulut et al.’s model of helical shaft uncoiling.

Further evidence from SEM images showed that the tip of the lancet exhibited a sharp, pointed structure with a clear helical axis (Figure 6B–D). This loose helical architecture was also apparent in *Gershwinia thailandensis* nematocyst samples, in which the free lancet displayed a pronounced helical groove along its length (Figure 6E).

During the early stages of eversion in the club-shaped microbasic p-mastigophores of *Gershwinia thailandensis*, LM imaging captured the emergence of the lancet from the capsule (Figure 6F,G), and in subsequent stages, a fully everted lancet remained attached to the shaft (Figure 6H,I). High-magnification images of isolated tentacular nematocysts revealed free lancets with sharp, narrow tips, which is consistent with a spearhead-like configuration (Figure 6K).

In the discharged oval microbasic p-rhopaloids of *Gershwinia thailandensis*, the lancet remained connected to the base of the tubule during initial discharge, with the lancet tip being the first structure to emerge from the capsule (Figure 6M). SEM images confirmed that the lancet retained its attachment to the shaft even after eversion, although the helical pattern was not as clearly visible in this nematocyst type (Figure 6N).

## 4. Discussion

In this study, three types of tentacular nematocysts—club-shaped microbasic p-mastigophores (Type 4), oval isorhizas, and oval microbasic p-rhopaloids—were identified in both *Gershwinia thailandensis* and *Morbakka* sp. A. While these nematocyst types were present in both species, significant differences in the sizes of the club-shaped microbasic p-mastigophores and oval isorhizas were observed. Previous studies have extensively reported nematocyst types in Cubozoan species, including single-tentacle species. For instance, Gershwin [43] and Bentlage and Lewis [5] documented nematocyst diversity in several box jellyfish species, which aligns with our findings. When compared to other species within the order Carybdeida, the nematocyst types observed here are consistent, although the number of nematocyst types can vary among species. For example, *Morbakka virulenta* has four types of nematocysts [5], whereas *Carybdea branchi* has only two [43,57]. Our study, in contrast, reports three nematocyst types in both *Gershwinia thailandensis* and *Morbakka* sp. A, with *Morbakka* sp. A having a relatively small population of oval microbasic p-rhopaloids. This variation in nematocyst size and composition may reflect species-specific ecological adaptations and functional differences, which warrant further investigation.

The club-shaped microbasic p-mastigophores identified in this study align with those reported in other species, such as *Malo filipina* [57], *Morbakka virulenta* [5], and *Morbakka fenneri* [43,58]. SEM characterization confirmed these nematocysts as Type 4 p-mastigophores, with spines consistently oriented away from the capsule. This structural feature is similar to those observed in other single-tentacle box jellyfish and members of the Irukandji clade, such as *Malo maxima* [59], *Gerongia rifkinae* [60], and *Malo kingi* [61]. These consistent morphological traits across species suggest a conserved function and evolutionary significance among these cnidarians.

One of the main challenges in this study was the identification of oval microbasic p-rhopaloids. The undischarged nematocysts, which are characterized by a V-shaped notch, were initially difficult to classify, as the discharged forms exhibited a single dilation along the shaft. Based on the classifications by Gershwin [43] and the updated descriptions by Östman [19], these nematocysts could be considered either p-mastigophores (undischarged) or microbasic euryteles (discharged). Nematocysts with a V-shaped notch at the distal end of the undischarged shaft are typically classified as p-mastigophores if the discharged shafts are cylindrical or as p-rhopaloids if the discharged shafts are lobed [43]. In this study, undischarged shafts consistently exhibited a V-shaped notch, and fully discharged samples showed the same feature at the end of the lancet. Additionally, discharged nematocysts displayed a single distal swelling with spines attached to the swollen area. Based on these observations and Gershwin’s classification, we identified these nematocysts as oval microbasic p-rhopaloids, with euryteles recognized as a subtype within the broader rhopaloid category.

The identification of isorhiza-type nematocysts in this study was relatively straightforward, although their sizes varied compared to other species within the order Carybdeida. Cnidomes can undergo changes throughout ontogeny, as seen in species like *Tomoya ohboya* [62] and *Chiropsalmus quadrigatus* [41], in which nematocyst composition shifts with body size. These changes may represent adaptations to prey-capture strategies. In species like *Chironex fleckeri*, it has been hypothesized that the ratio of nematocyst types and venom composition evolves as the organism matures, indicating potential ontogenetic changes in both nematocyst structure and venom characteristics [63]. Larger species, such as *Chironex fleckeri*, tend to have a higher proportion of mastigophores, likely reflecting their need to capture larger prey [53,54].

This study presents the first detailed observation of tubule spine characteristics in *Gershwinia thailandensis* and *Morbakka* sp. A, two Carybdeida box jellyfish species from Thai waters. Notably, all three nematocyst types—club-shaped microbasic p-mastigophores, oval isorhizas, and oval microbasic p-rhopaloids—exhibited consistent tubule patterns and spine arrangements when fully discharged. Comparisons with Hydrozoa species reveal distinct differences. For example, isorhiza nematocysts in *Obelia dichotoma* display different spine shapes and arrangements [64]. Similarly, while *Apolemia* sp. exhibits arrow-shaped spines in a helical pattern, the spine size and tip shape differ [19]. Additionally, heterotrichous anisorhizas in *Physalia physalia* (Hawaiian bluebottle) possess claw-shaped spines with sharper tips, although they share similarities in spine arrangement [27]. These comparisons suggest species-specific structural adaptations among cnidarians.

When compared to other Cubozoan species, such as *Chironex fleckeri* [35], the spine morphology of the discharged microbasic mastigophores observed in this study more closely resembles the isorhiza nematocysts in *Apolemia* sp. [19] and heterotrichous anisorhizas in *Physalia physalia* [27], rather than *Chironex fleckeri*. Although the spine shape and arrangement in the heterotrichous microbasic euryteles of *Carybdea alata* differ [40], the tapering tubule diameter toward the distal end, as seen in our findings, aligns with these observations. This supports the hypothesis that tubule and spine arrangements vary among species, potentially reflecting evolutionary adaptations and functional roles within Cnidaria. These structural differences may influence the penetrative capabilities of nematocysts, contributing to interspecies variations in toxicity. According to Gershwin (2006) [43] nomenclature, all nematocyst types identified in this study are heterotrichous, with tubules covered in spines of varying sizes. Notably, the spines near the proximal tubule, adjacent to the capsule, differ in size from those at the distal end, further emphasizing the complexity of nematocyst morphology and function.

The operculum structure observed in the club-shaped microbasic p-mastigophores (Figure 1G) and oval isorhizas (Figure 2D,E) of *Gershwinia thailandensis* appears triangular, which aligns with the synapomorphic presence of an operculum in Medusozoa, as detailed by Reft and Daly, 2012 [65]. This triangular operculum contrasts with findings in other Cubozoan species like *Chironex fleckeri*, in which Rifkin and Endean, 1983 [35], reported that mastigophores and euryteles have triangular opercula, while isorhizas possess a circular operculum. Moreover, this study contributes to understanding nematocyst apical structures by showing that the isorhizas of *Gershwinia thailandensis* feature a tubule tip resembling a twisted propeller, similar to that seen in stenotele nematocysts of *Hydra attenuata* (Hydrozoa) [66]. These observations suggest potential structural variation across species, likely contributing to differences in nematocyst function and prey capture mechanisms. Furthermore, Reft and Daly (2012) proposed that the operculum is a synapomorphy for Medusozoa, whereas apical flaps characterize Anthozoa. However, their findings also identified an apical cap in some non-actiniarian Anthozoans, which opens further questions about the evolutionary divergence of these apical structures. In *Gershwinia thailandensis*, we observed no such apical cap, reinforcing the structural diversity within Cnidaria nematocysts and the significance of operculum morphology for taxonomic identification and understanding of nematocyst function.

In this study, we observed a dominant shaft structure, referred to as the “lancet”, in both club-shaped microbasic p-mastigophores and oval microbasic p-rhopaloids in *Gershwinia thailandensis* and *Morbakka* sp. A. The lancet first appears during the initial stage of discharge and remains attached to the distal shaft in fully discharged nematocysts (Figure 6). This finding is consistent with previous studies on eurytele nematocysts in *Carybdea alata*, in which the lancet remained intact in rapidly fixed tentacle sections but was absent in preparations subjected to prolonged isolation or delayed fixation [40]. In our study, rapid fixation retained the lancet, while longer processing times led to its absence, reinforcing the importance of fixation timing for preserving nematocyst structures.

The formation and function of lancet-like structures have been documented in Anthozoa and Hydrozoa, in which they are often termed “darts” [67,68,69]. In cubozoans, Yanagihara [40] suggested that the lancet of *Carybdea alata* may function similarly to the stylets in Hydra, aiding in tubule penetration by perforating prey tissue. Our findings support the hypothesis that lancet structures are not unique to *Carybdea alata* but may be common among heterotrichous microbasic euryteles, as we identified lancets in both p-mastigophores and p-rhopaloid nematocysts.

Nematocyst identification through skin scraping is a widely used method for collecting jellyfish specimens from sting cases, providing valuable insight when combined with clinical presentation and jellyfish identification for accurate diagnosis and treatment [55,70,71]. In Thailand, severe envenomations are commonly caused by *Chironex* spp., *Morbakka* spp. (Cubozoa), and *Physalia* spp. (Hydrozoa), with each species exhibiting distinct nematocyst features [55]. Our study contributes to this knowledge by further characterizing the nematocyst features of box jellyfish, which can help link specific nematocyst characteristics to the symptoms they cause. Expanding this understanding will enhance species identification and improve treatment strategies for jellyfish stings in future research [72].

## 5. Conclusions

This study represents the first detailed examination of nematocyst types in the tentacles of *Gershwinia thailandensis* and *Morbakka* sp. A found in Thai waters. Three nematocyst types—club-shaped microbasic p-mastigophores (Type 4), oval isorhizas, and oval microbasic p-rhopaloids—were identified using light microscopy and scanning electron microscopy. The nematocyst types varied in size and population density within the tentacles of both species. The pattern and shape of the tubule spines observed in these species may be linked to their evolutionary history and potential injury mechanisms. The presence of triangular opercular flaps also suggests possible evolutionary relationships and developmental processes within the phylum Cnidaria.

The complexity of nematocyst structures, both in their undischarged and discharged states, has been explored in various Cubozoan species. This study contributes to our understanding of nematocyst structure and morphology, enhancing our knowledge of phylogenetic relationships. Furthermore, these findings may be valuable for medical professionals in Thailand who need to identify sting samples in cases involving various species and for future research aimed at classifying box jellyfish species.

However, due to limited sample availability and preservation constraints, further quantification is not feasible. Our study primarily focused on morphological and structural analysis, and we will consider quantitative comparisons in future research when more samples are accessible. 

## Figures and Tables

**Table 1:** Capsule size of different undischarged nematocyst types in *Gershwinia thailandensis* and *Morbakka* sp. A.

Species	*Gershwinia thailandensis*		*Morbakka* sp. A	
Type of Nematocyst	Capsule Length (µm)	Capsule Wide (µm)	Capsule Length (µm)	Capsule Wide (µm)
club-shaped microbasic p-mastigophores Type 4 Range: Min–Max (n)	58.25 ± 6.56 ^ab^	17.07 ± 1.80	71.04 ± 10.30 ^ab^	17.49 ± 2.40
	43.29–77.22 (n = 350)	12.97–24.78	51.78–89.80 (n = 244)	13.00–23.12
oval isorhizas Range: Min–Max (n)	45.71 ± 5.33 ^cd^	34.98 ± 3.44	72.74 ± 7.10 ^cd^	38.58 ± 3.47
	31.57–64.80 (n = 205)	22.53–40.74	56.84–81.38 (n = 46)	29.93–43.89
oval microbasic p-rhopaloids Range: Min–Max (n)	44.28 ± 2.45	22.19 ± 1.69	42.74 ± 6.06	19.56 ± 1.33
	35.96–50.38	18.63–29.47	34.10–51.21	17.68–20.91
	(n = 245)		(n = 5)	

Note: Statistical differences in the same nematocyst types between *Gershwinia thailandensis* and *Morbakka* sp. A are denoted by different letters (a–d), with significance at *p* < 0.01.

## Data Availability

The raw data supporting the conclusions of this article will be made available by the authors upon request.
